# Healthcare Information Avoidance in the Context of Caring for a Child with a Serious Illness

**DOI:** 10.3390/children12111464

**Published:** 2025-10-29

**Authors:** Tiina Jaaniste, Shujauddin Mohammed, Sue Cowan

**Affiliations:** 1 Department of Palliative Care, Sydney Children’s Hospital Randwick, Sydney, NSW 2031, Australia; shujauddin.mohammed@student.unsw.edu.au (S.M.); sue.cowan@health.nsw.gov.au (S.C.); 2 School of Clinical Medicine, University of New South Wales, Kensington, NSW 2052, Australia

**Keywords:** avoidance, caregiver, information, palliative care, pediatric

## Abstract

**Highlights:**

**What are the main findings?**

• Caregiver healthcare information avoidance may occur as a short-term coping strategy.
• Caregiver healthcare information avoidance is likely to be unhelpful as a pervasive, long-term behavior.

**What are the implications of the main findings?**

• Healthcare professionals should identify the reasons for caregiver healthcare information avoidance.
• Preparation and planning should occur before healthcare professionals share difficult information with families.

**Abstract:**

Caregivers of a child with a serious medical condition are often confronted with difficult and stressful medical information. While they commonly seek out health-related information to better care for their child and help with their decision-making, sometimes caregivers engage in healthcare information avoidance. Healthcare information avoidance is the decision to prevent or delay the acquisition of available, but potentially unwanted, health-related information. We begin by defining the construct of healthcare information avoidance and exploring key theoretical frameworks that illuminate its underlying mechanisms including emotion regulation theory, attentional and cognitive models, approach-avoidance coping strategies, and dispositional theories. A lack of validated measures to assess caregiver healthcare information avoidance was noted as contributing to the dearth of empirical work in this area. Common areas of caregiver healthcare information avoidance were identified at various points throughout the pediatric palliative care illness trajectory. The review concludes with directions for future research and practical recommendations for clinical care, highlighting the importance of identifying the occurrence and reasons for caregiver information avoidance as well as optimizing approaches to information provision.

## 1. Introduction

Caregivers—defined here as parents and others with primary responsibility for a child’s care—are often faced with difficult and stressful medical information when a child has a serious health condition This information may include test results, diagnoses, current symptoms and possible developments, treatment options, medications, comfort measures, available services, and the overall illness trajectory and prognosis. At each turning point in the child’s illness trajectory, they are likely to be confronted with new information and decisions. Healthcare professionals (HCPs) generally aim to empower families by keeping them well-informed [1]. Although caregivers often desire healthcare information to better understand and care for their child, sometimes they prevent or delay the acquisition of information, engaging in healthcare information avoidance. Available information may be potentially distressing or overwhelming, and at times, caregivers may be more inclined to avoid receiving such information [2].

There are many possible reasons for why caregivers may engage in healthcare information avoidance, ranging from believing that the available information may not be helpful or credible, through to fearing that the information is accurate but undesirable [2,3]. The current review will explore the theoretical and empirical literature pertaining to information avoidance in the context of pediatric healthcare, particularly in the context of caring for a seriously ill child. A detailed description of the construct of healthcare information avoidance will be provided. This will be followed by an overview of key theoretical perspectives that shed light on information avoidance in the context of caring for a child with a serious medical condition. Methods used to assess information avoidance in the healthcare context will be described. Healthcare information avoidance behaviors in the pediatric palliative care context will be considered, noting how these may occur at different time points in the illness trajectory. The review will conclude with directions for future research and tentative recommendations for clinical practice.

## 2. Construct of Information Avoidance in the Context of Healthcare

Information avoidance may be defined as the decision to prevent or delay the acquisition of available, but potentially unwanted, information. Such information avoidance may be active or passive. Active avoidance involves a direct behavior intended to disengage with information such as refusing a healthcare appointment where a diagnosis is likely to be discussed or removing oneself from such a conversation [2]. Passive avoidance occurs through inaction such as not asking questions that may reveal undesired information. Passive avoidance may be difficult to identify, requiring insight into an individual’s intentionality or motivation [3,4].

Information avoidance may be either a behavioral or cognitive process [5]. A behavioral process occurs when the individual takes physical measures to prevent information acquisition (e.g., by not opening a letter or not engaging in a discussion with a HCP who may be ready to provide information). Cognitive avoidance processes may occur when an individual does not focus on or attend to potential sources of unwanted information.

Within the context of a serious medical condition, information avoidance may sometimes be wrongly interpreted by clinicians as indicating that the individual is in denial or not coping well [6]. However, in some situations, information avoidance may be a coping strategy used by individuals to help them to manage information overload or prevent them from feeling overwhelmed [7]. Healthcare information avoidance may be helpful in the short-term, enabling the individual to process information in their own time, or enabling them to manage their emotions before receiving additional, potentially distressing information. It can provide individuals with a sense of control, enabling them to filter and manage the timing and amount of new information received [7], and serve as a strategy to manage their negative emotions [8]. However, an over-reliance or prolonged use of information avoidance in the healthcare context may be problematic. In the broader healthcare context, it can impair decision making and result in poorer health outcomes [9,10].

The available literature on healthcare information avoidance focuses predominantly on the avoidance of one’s own healthcare information. However, in the case of pediatric healthcare, it is typically the parent who is responsible for receiving or accessing information about their child’s condition. Caregivers may at times engage in proxy healthcare information avoidance, meaning that they avoid information pertaining to their child’s healthcare. Such proxy avoidance may occur for similar reasons that individuals avoid health-related information about themselves, for example, if the information is likely to be distressing or perceived to lack credibility. Caregivers may also have additional considerations about whether the information would be in the best interests of the child. Anecdotally, caregivers may avoid healthcare information about their child if they think that it is likely to cause distress and add to the child’s burden but offer no tangible benefit. However, to our knowledge, there have been no studies on caregiver information avoidance and perceived information utility.

It has been suggested that healthcare information avoidance may be considered as a continuous, rather than dichotomous, construct, whereby it is possible to engage in greater or lesser degrees of information avoidance [11]. Information avoidance may be extensive and persistent or it may be partial or short-term. Furthermore, information avoidance and seeking behaviors can co-occur, whereby individuals may avoid information on a certain topic whilst seeking information on other topics. Within the literature, there has been little acknowledgement of the broad spectrum of healthcare information avoidance and the contextual settings in which greater avoidance occurs.

## 3. Theories of Information Avoidance

**Figure 1:**
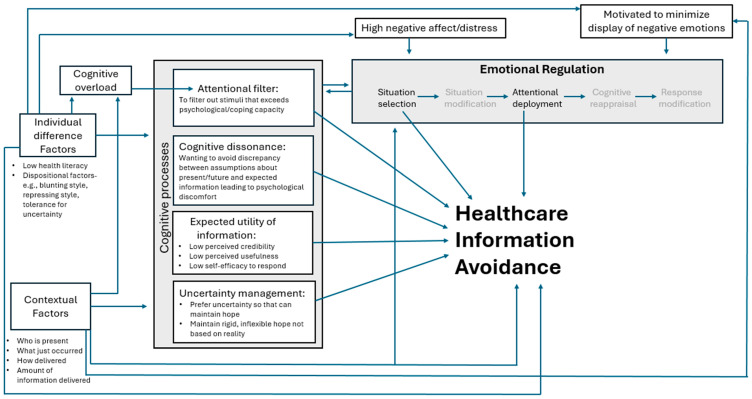
Key factors and theories that may impact caregiver healthcare information avoidance.

A range of theories, from differing perspectives, may be applied to account for the processes involved in information avoidance [2,12]. These theories focus on (a) emotion regulation, (b) attentional and cognitive processes, (c) approach-avoidance models of coping, and (d) stable, dispositional factors. Each of these theoretical perspectives are examined in more detail below. Figure 1 graphically depicts how some of the key theories and related factors may impact on healthcare information avoidance.

*Emotion regulation theory*. This theory holds that individuals utilize conscious and unconscious strategies to influence the emotions that are experienced [13]. Notably, emotion regulation approaches may be intrinsic, where an individual seeks to regulate their own emotions, or extrinsic, such as when other people like HCPs seek to help patients and families to better manage difficult emotions [14]. Given that parents of children with a serious illness have higher rates of post-traumatic stress disorder (PTSD) related to their child’s medical trauma [15], emotion-regulation avoidance strategies are particularly common in these individuals, this being part of the diagnostic criteria of PTSD. Emotion regulation may occur at various phases of an experience [16], namely: (i) situation selection, (ii) situation modification, (iii) attentional deployment, (iv) cognitive reappraisal, and (v) response modulation. Each of these phases of emotion regulation are described in more detail below, and illustrative examples are provided for how individuals may respond to available information. Notably though, information avoidance is most likely to occur in the ‘situation selection’ or ‘attentional deployment’ phases.

(i) Situation selection. An individual may shape their environment to limit exposure to unwanted information. For example, a parent might leave a conversation with an HCP if they believe that undesired information may be shared, or they may only read online resources that are likely to align with their views.
(ii) Situation modification. To lessen negative emotional impact, individuals may adjust situations such as inviting a partner/friend for support or engaging in calming activities (e.g., going for a walk, praying) before receiving difficult news. These strategies may be used to minimize information avoidance.
(iii) Attentional deployment. This involves shifting attention away from potentially distressing information to lighter topics for emotional relief. Conversely, individuals may engage in rumination, or a perseverative focus on the information and associated thoughts and feelings, which is often unproductive and may intensify negative emotions [17,18].
(iv) Cognitive reappraisal. Reframing the meaning of a situation can alter emotional responses. For example, a parent may shift from viewing a failed intervention as a devastating loss to seeing it as a relief for their child from a burdensome treatment.
(v) Response modulation. This form of emotion regulation occurs late in the emotion-generation process and involves altering physiological, experiential, or behavioral responses associated with an emotional situation [14]. For example, intense exercise may be used to decrease the physiological and experiential aspects associated with the negative emotion. [14]. Another form of response modulation is expressive suppression—choosing not to show negative emotions after receiving bad news. HCPs may misinterpret this as denial rather than attempts at maintaining some control over where and when negative emotions are experienced. Nevertheless, prolonged suppression can lead to negative emotions “leaking” and manifesting in other contexts [14,19].

*Attentional and cognitive theories.* Humans have finite attentional resources for receiving incoming stimuli or information [20], which is further reduced under stress [21]. Individuals choose where to direct their attention [21], and during stress may consciously avoid new information or may unconsciously fail to process it. Cognitive biases, such as shaped by perceived threat, relevance, and interest, may influence attention and avoidance [22]. For example, Internet searches are often biased based on interests, values, beliefs, and educational level.

Cognitively focused theories of information avoidance assume that decision making is a predominantly rational process [23], with individuals weighing up options based on specific criteria. Relevant theories that may impact on information avoidance include expected utility theory [24], uncertainty management theory [12,25], and cognitive dissonance theory [26].

The expected utility theory suggests that individuals act rationally, choosing the option they believe offers the greatest benefit [24]. Although rooted in economics [23], the theory has potentially useful, but less explored, clinical applications. Individuals make choices about whether to receive available information based on whether receiving or not receiving the information affords greater benefit. For example, caregivers may seek information if it improves their child’s care, but avoid it if it is distressing and offers no clear advantage to the child or family. Moreover, if individuals lack self-efficacy or confidence in their ability to respond to available information, they may be less likely to feel empowered by receiving it and more likely to avoid information [10].

Uncertainty management theory, a more specific example of the expected utility theory, suggests that information behavior can reduce, increase, or maintain levels of uncertainty [12]. In some cases, individuals may prefer to maintain uncertainty rather than receive information that eliminates it [25]. For example, a parent of a sick child may avoid definitive negative information about their child’s diagnosis or prognosis, but rather maintain a degree of uncertainty to preserve hope, even if that hope is no longer supported by evidence.

The cognitive dissonance theory suggests that information conflicting with an individual’s beliefs causes psychological discomfort that they are motivated to reduce [26]. To minimize this discomfort, individuals may avoid information that conflicts with their existing beliefs and contributes to this cognitive dissonance [27,28,29]. For example, a parent expecting their child to start school and develop in ways similar to other children may avoid news of a serious and progressive medical condition that will impact their child’s ability to develop normally, thus using avoidance to ease the uncomfortable cognitive dissonance.

*Approach-avoidance models of coping.* These models of coping have existed in the psychological literature for over a century [30,31,32] and have more recently been framed in various ways (e.g., dual process model [33], parallel process model [34]). Both approach and avoidant responses may at times be beneficial [11]. For example, the bereavement literature highlights that grieving individuals may switch between focusing on loss and on restoration [35,36]. Similarly, a dual-process framework has been used to suggest that hope for a cure and awareness of poor prognosis can co-exist in caregivers of a child with a serious medical condition [37,38]. Some caregivers may appear focused on hoping for a cure but occasionally ‘visit’ the ‘reality’ of their child’s prognosis, where the likelihood of a cure is low. Katz et al. (2021) emphasized the importance of clinicians acknowledging this duality, rather than assuming that hope precludes awareness of illness severity [38].

In healthcare information provision, an approach-avoidance model of coping explains how individuals may shift between seeking and avoiding information based on coping needs. Seeking information supports informed decision-making, while avoidance may offer relief from emotional and cognitive strain [6,33].

*Dispositionally based theories.* Dispositional theories suggest that stable, personality features may influence an individual’s tendency to avoid information. Two related personality styles that have a long history in the literature are monitoring vs. blunting [39] and sensitizing vs. repressing [40]. Individuals classified as ‘blunters’ prefer distraction and avoid details, especially in stressful situations [39]. In contrast, ‘monitors’ seek detailed information and scan for risk. Similarly, ‘repressors’ minimize or avoid threatening information, pushing away anxious thoughts, whereas ‘sensitizers’ actively seek out information and focus on potential threats.

More recently, an individual’s tolerance for uncertainty has been considered in relation to their proclivity to seek or avoid information [12]. Individuals with low tolerance for uncertainty may seek information, striving to achieve a sense of certainty on an issue. Once a sense of certainty or closure is achieved, they may avoid further information that could disrupt their understanding [41].

Research has shown that tailoring information to patient dispositions or preferences can reduce anxiety levels [42,43]. However, dispositional categorization may be overly simplistic and fail to recognize that individuals may respond differently depending on situational and contextual demands [44,45].

*Cultural factors.* Various cultural groups may tend to respond to information differently, with some groups less likely to actively seek information from HCPs [46]. Less is known about whether some cultural groups are more likely to avoid available information. Historically marginalized groups may have intergenerational trauma, contributing to a greater mistrust of HCPs and the information that they have to offer [47].

## 4. Assessment of Healthcare Information Avoidance

Our current understanding of parental information avoidance in the pediatric palliative care context is limited by the lack of suitable assessment tools to assess the construct. Most existing adult healthcare information avoidance measures have focused on the avoidance of one’s own healthcare issues, rather than in the context of caring for a child. For example, the Information Avoidance Scale [5], Health Information Orientation Scale [48], and Threatening Medical Situations Inventory [49] all assess information avoidance behavior among adults with respect to their own actual or potential healthcare situations.

A number of studies have also used study-specific vignettes or hypothetical questions to enquire about how individuals would respond in certain situations. For example, one study used a vignette to determine that 39% of their healthy adult sample (*n* = 2974) would prefer not to know their chance of developing cancer [4]. Another study used specific questions about health-related information avoidance, finding that 30% and 34% of the respondents preferred to avoid receiving information about diabetes and colorectal cancer, respectively [50]. The lack of validated measures for assessing parental healthcare information avoidance, particularly with the sensitivity for assessing changes over time, is likely to be contributing to the dearth of quantitative studies in this field.

## 5. Information Avoidance in the Pediatric Palliative Care Context

The context of caring for a child with a serious or life-limiting condition entails the need for many caregiver decisions about the child’s management and care options. Caregivers need the provision of accurate and understandable healthcare information in order to make informed decisions.

**Figure 2:**
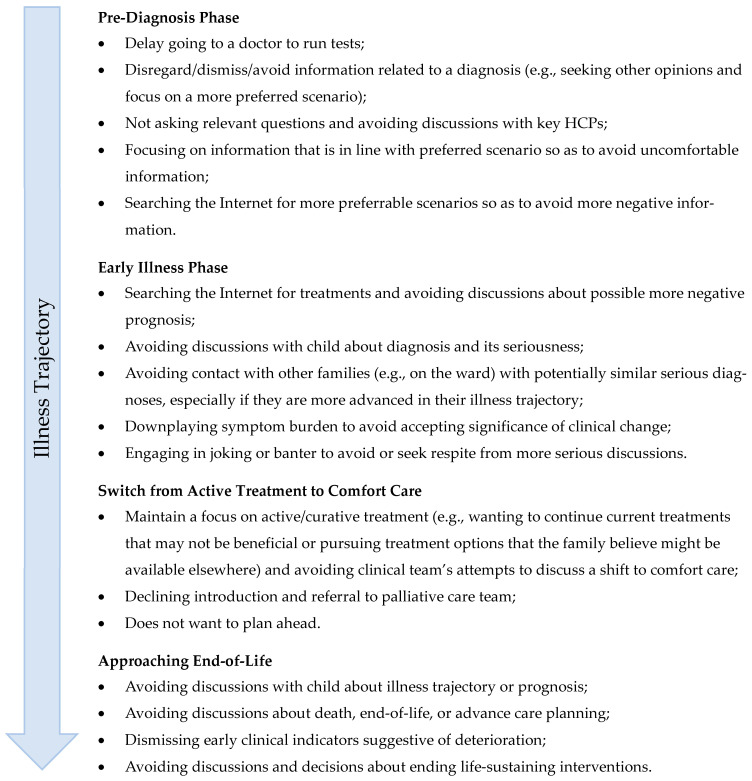
Types of caregiver information avoidance scenarios that may occur at key points of the child’s illness trajectory.

Although data are currently lacking, anecdotally, it is not uncommon for caregivers of children with a serious medical condition to avoid, in some situations, receiving healthcare information that is available to them. Figure 2 highlights common examples of clinical interactions that may be reflective of caregiver information avoidance.

Furthermore, HCPs need to take care that their own actions and possible discomfort are not inadvertently perpetuating the family’s information avoidance. Families and HCPs may sometimes collude in a hopeful narrative, perhaps longer than is helpful to do so. In an era of many available clinical trials, HCPs may sometimes feel more comfortable travelling with the family between successive treatments and trials, even as these become increasingly less likely to provide tangible benefits. As families cling to the hopeful narrative, they may avoid or dismiss information that suggests something to the contrary.

## 6. Future Research Directions

Research into information avoidance in the context of caring for a child with a serious or life-limiting illness relies on the accurate assessment of this construct. There is a need for the development of measures designed to assess healthcare information avoidance amongst caregivers of children with serious medical conditions. Drawing on measures of information avoidance developed predominantly in adult healthcare contexts [5,48,49,51] may lack the nuances specific to the pediatric palliative care context. Moreover, there is a need to better understand the specific contexts in which caregivers of children with a serious medical condition may avoid healthcare information and their reasons for such avoidance. Furthermore, greater attention is needed in the development and evaluation of strategies to share complex and potentially abstract information with caregivers who have less education or who are neuroatypical.

Longitudinal research would be useful to better understand the use of parental information avoidance throughout a child’s illness trajectory. Moreover, it would improve our understanding of individual’s switching between information avoidance and information seeking behaviors. One may postulate that an individual’s capacity to switch between these approaches may be indicative of optimal coping, ensuring that necessary information is obtained, but that they are able to protect themselves from feeling too overwhelmed at times when they are not ready to receive more information. However, longitudinal data on this issue are currently lacking.

## 7. Clinical Directions for Managing Information Avoidance

Caregivers of children with a life-limiting condition receive healthcare information from many different sources. The palliative care team is typically one of many teams involved, with the primary medical teams often providing the first line of information about diagnosis and prognosis. Caregiver information avoidance may occur in some specific contexts, or it may pervade across all areas. Well-coordinated and well-administered information provision strategies are needed to ensure that families have access to the information they need, in a way that takes into account their capacity to absorb and process information, enabling them to prepare for future care needs and to make effective care decisions. When healthcare information avoidance behaviors are noted, a range of coordinated strategies may be implemented to best support the caregiver. These involve identifying the pervasiveness of the avoidance, ascertaining the reasons for avoidance, considering how best to prepare the individual to receive information, and ensuring that measures are in place to support the individual after information is provided. These approaches will be considered in more detail.

*Recognizing the pervasiveness of information avoidance.* It is helpful for HCPs to recognize what contexts caregivers are avoiding information in. To overlook these avoidance behaviors, and to attempt to provide healthcare information in the face of active avoidance, may negatively impact on the rapport between the healthcare provider and family member [52]. Moreover, an understanding of the extent or pervasiveness with which an individual avoids healthcare information enables the provider to recognize whether the individual occasionally ‘visits’ the reality of the situation and is in certain situations willing to receive healthcare information [38]. HCPs may seek to support this coping style by trying to understand which contexts the parent is likely to be willing to ‘visit’ the difficult reality of the situation and receive information. Given that complete and pervasive avoidance of healthcare information is likely to be problematic, preventing individuals from making informed healthcare decisions, HCPs may encourage the individual to think about how they might most comfortably ‘visit’ the difficult information and what contextual factors may best support them in doing so.

*Identifying the reasons for avoidance.* It is helpful to ascertain why caregivers may be avoiding healthcare information and what purpose such information avoidance may be serving. For example, has the caregiver already received a lot of information that they are still processing? Are they trying to regulate their emotions, which the available information potentially threatens? Does the available information potentially threaten how the caregiver views themselves and their child? Does the information potentially challenge the thing that the caregiver most hopes for? Does the caregiver recognize the value or relevance of the available information? If avoidance is recognized as a short-term coping strategy, where possible, caregivers should not be forced to receive information if they are clearly indicating their unreadiness to do so [52].

If an HCP suspects that a caregiver may be avoiding information, it would be helpful to discuss this possibility with them and explore possible reasons for their behavior. Incorrectly identifying avoidant coping responses as denial may result in HCPs reiterating the negative information that the individual may already be overwhelmed by and seeking respite from [6]. A sensitive and gentle discussion may be needed to help recognize the caregiver’s reasons for information avoidance, with acknowledgement of any short-term benefits of such avoidance. This can then be followed by consideration as to what would help to move the individual toward greater readiness to receive information.

*Preparation and readiness to receive information.* When individuals have the opportunity to prepare themselves for a difficult situation, they may be in a better position to utilize their coping strategies or seek out appropriate supports [53,54]. Similarly, it may be beneficial to fore-warn individuals before sharing difficult healthcare information. This may enable them to draw from their coping resources and maintain a sense of control in the situation [55]. This aligns with the anxiety treatment literature, which highlights the importance of minimizing threat avoidance behaviors, which over time offer minimal benefits and limit more adaptive coping [8]. There is recognition that exposing individuals to threat stimuli should be carried out in a planned and staged way to avoid causing further trauma [56]; similarly, individuals should be exposed to stressful information in a carefully planned and controlled way. A qualitative study with adult palliative care patients found that the provision of difficult information when an individual was not ready for it could be harmful for the individual’s psychological welfare and their relationship with the HCP providing the information [52].

Table 1 outlines some key steps and specific strategies when preparing to give individuals difficult information. These strategies are best embedded within a multidisciplinary framework. Whereas a clinical psychologist might address adaptive coping approaches, a social worker may focus on social support networks, and a chaplain may discuss where the individual finds spiritual peace. Thus, each of these approaches may contribute to the individual’s capacity to receive difficult information.

## 8. Conclusions

When caring for a seriously unwell child, healthcare information may help caregivers in making informed decisions about the care of their child. Nevertheless, caregivers may sometimes engage in healthcare information avoidance. A range of theoretical perspectives may account for aspects of information avoidance behavior. While short-term healthcare information avoidance may serve as a beneficial coping strategy, as a long-term approach, it may compromise a parent’s capacity for making informed healthcare decisions. A range of clinical strategies are suggested to assist in preparing individuals to receive difficult information.

## Figures and Tables

**Table 1:** The CACARAF steps for preparing to deliver difficult healthcare information to caregivers.

Key Steps	Considerations	Illustrative Comments
**C**. Assess individual’s capacity for **Coping** with further information provision	• Has anything just happened that might impact on the individual’s short term coping capacity (e.g., clinical crisis, recent difficult information already received)? • What has previously helped this individual to cope with difficult situations? • Support the individual to utilize available coping resources.	“Who or what do you turn to for support during tough times?” “Is there anything that you would find helpful…”
**A.** Identify individual’s current **Affective** state	• Is the individual’s affective state likely to impact on their capacity to process and absorb the information? • Is the individual’s negative affective state transient (e.g., in response to acute stressor) and likely to improve or persistent?	“It is understandable that you are quite shaken by the things that happened; there has been a lot for you to take in. Let us talk more tomorrow, and I can explain things more and answer any questions.”
**C.** Provide **Choice** regarding the delivery of the information provision	• Where possible provide appropriate choices regarding the location, timing and circumstances of information provision.	“Would you find it helpful to bring someone with you to the next meeting?” “Would you prefer that we talk here or in a room away from the ward?”
**A.** Inform individual of the nature of the **Available** information	• Indicate the topic of information (e.g., test results, prognosis) and provide some indication of whether information is likely to be perceived as negative.	“Some test results have come back. The results might not be all that we hoped for.”
**R.** Enquire about individual’s perceptions of their **Readiness** for information	• Request permission to provide information before proceeding.	“Would it be OK with you if I give you some information about the different options?”
**A.** Consider the **Amount** of information that is useful to provide on the one occasion	• Prioritize which information should be given first and whether some information can wait for a later occasion. • Be guided by the individual’s verbal and non-verbal communication as to whether they have capacity to receive further information.	“There is a lot to take in. We do not need to talk about it all today. For now, we could just focus on…”
**F.** Ensure appropriate **Follow-up**	• Will they have sufficient opportunities to ask questions when they think of them? • Will they have someone (e.g., partner, friend, social worker) to help them discuss and process the information afterwards? • If the new information is incongruous with their prior hopes, discuss and provide support in shifting the focus of their hope.	“If more questions come to mind, write them down and we can talk about them tomorrow” “After you leave here, is there someone you can talk this through with?”

## Data Availability

Not applicable.

## Abbreviations

The following abbreviation is used in this manuscript:

HCP	Healthcare professional
